# Prolonged Second Stage of Labor and Levator Ani Muscle Injuries

**DOI:** 10.5539/gjhs.v7n1p267

**Published:** 2014-09-28

**Authors:** Vajihe Marsoosi, Ashraf Jamal, Laleh Eslamian, Sonia Oveisi, Shokohossadat Abotorabi

**Affiliations:** 1 Perinatology Division, Department of Obstetrics and Gynecology, Shariati Hospital, Tehran University of Medical Sciences, Tehran, Iran; 2 Department of Obstetrics and Gynecology, Faculty of Medicine, Tehran University of Medical Sciences, Tehran, Iran; 3 Metabolic Diseases Research Center, Medical School, Qazvin University of Medical Sciences, Qazvin, Iran; 4 Department of Obstetrics and Gynecology, Faculty of Medicine, Qazvin University of Medical Sciences, Qazvin, Iran

**Keywords:** ultrasound, labor, puborectalis avulsion, levator ani, pelvic floor

## Abstract

**Objective:**

To determine the effect of pregnancy and vaginal delivery on the pelvic floor and levatorani morphology and function.

**Methods:**

Design. Cross-sectional study. Setting. Tertiary care teaching hospital. Population. 75 primigravid women were recruited for assessment at 6 weeks postpartum compared with 25 nulliparous women. Hiatal morphology and levator ani muscle avulsion were assessed by 4-dimensional translabial ultrasound examination. The volume achievement obtained by ultrasound was performed in supine position with empty bladder at rest, on maximum Valsalva maneuver, and on maximum pelvic floor muscle contraction. Main Outcome Measures. Hiatal diameter and area were measured at the plane of minimal hiatal dimension as defined in the midsagittal plane and Levator avulsion was assessed.

**Results:**

There were significant differences in hiatal area morphology at rest, on Valsalva maneuver and during contraction of muscles among the study groups, but there was no difference in pelvic diameter at rest, on Valsalva maneuver, and during contraction. There were 21 cases of puborectalis avulsion (42%) with no significant difference between non-progressive labor (8 cases) and Normal Vaginal Delivery (NVD) (13 cases) groups.

**Conclusions:**

The results of the present study showed that non-progressive labor is the main risk factor for pelvic muscle injuries, indicating the necessity of a better management and timely cesareans in women with prolonged second stage of labor.

## 1. Introduction

Pelvic floor is a general expression that includes all the muscles, connective tissues, and other structures located in the pelvic cavity; a network of muscles with crucial role in maintaining and securing an appropriate functioning of the organs located in the pelvis. Normal Vaginal delivery (NVD) is a major predisposing factor for the development of vaginal and pelvic floor disorder such as genital prolapse and urinary or anal incontinence. The stretch of levator ani muscle throughout delivery causes hiatal opening during movement of the fetus (Cassadó Garriga et al., 2011).

The rupture of levator ani muscle is especially presented in puborectalis muscle. An irreversible change occurs following the vaginal delivery which can cause levator ani dysfunction (Cassadó Garriga et al., 2011). The development of urinary incontinence after delivery may mostly be due to broad damage or denervation of the pelvic floor (Leeuw, Vierhout, Struijk, & Hop, 2001).

Levator ani and puborectalis are the major contributors of levator hiatus which has an important and central role in supporting the control of urination and defecation and that the rupture of this muscle especially in the inferior pubic ramus and the pelvic wall, due to vaginal delivery, can cause hiatal opening (Dietz & Shek, 2008).

Hiatal diameter enlargement especially occurs in the second stage of labor and during crowning of the fetal head in older primiparous women, this rupture is to some extent identifiable by digital examination (Dietz & Shek, 2008).

Prolonged second stage of labor is a common condition in all labor wards. The obstetrician has three options; do a cesarean section, achieve a vacuum extraction or deliver by forceps (Geirsson & Eggebø, 2012). The hiatal opening varies among different patients due to the dissimilarity of variable stretches of muscles during pregnancy and delivery (Svabík, Shek, & Dietz, 2009). The rupture is now detectable by clinical examination, urodynamic tests, anorectal physiology tests, video cyst urography, and dynamic cyst urography. Dynamic MRI is not cost effective whereas the application of sonography in assessing the pelvic floor is gradually on rise due to its accessibility and effectiveness. Sonography is an effective modality because of the absence of ionizing rays, easy application, cost effectiveness, and low time consuming (Santoro et al., 2011). The aim of this study was to determine the muscle changes of pelvic floor by sonography following pregnancy and vaginal delivery.

## 2. Materials and Methods

This was a cross-sectional study performed on women referred to the gynecology clinics of Dr Shariati Hospitals in Tehran (Iran) in 2012, in whom the pelvic floor muscles was assessed using a Siemens sonography device. The inclusion criteria were: 1) singleton pregnancy, 2) a minimum age of 18 years, and 3) lack of previous pregnancies of more than 20 weeks of gestation. All potential participants were contacted after delivery of necessary information over the study and requested to be examined with ultrasound approximately a month after the delivery. Also, a six-week postpartum appointment was arranged for those interested in participating in the study plus 25 nulliparous women, referred to the gynecology clinics, were also included. The participants were enrolled in the study after obtaining informed consent.

All women were subjected to an introital 3D and 4D ultrasonography using Siemens Antares System (Siemens Healthcare, USA) with C7F2, 3D and 4D ultrasound transducer with the acquisition angle is set at 85° to include the entire levator hiatus(Steensma, 2009). The volume achievement obtained by ultrasound was performed in supine position with empty bladder at rest, on maximum Valsalva maneuver, and on maximum pelvic floor muscle contraction.

The operator was blinded to clinical data, with the patient’s abdomen covered by a sheet. Hiatal diameter and area were measured at the plane of minimal hiatal dimension as defined in the midsagittal plane. Levator avulsion was diagnosed whenever a defects mostly occur as a detachment of the puborectalis on the anteromedial part of the attachment of the levator ani to the pubic bone. These defects can be either unilateral, left or right, or bilateral (Steensma, 2009). In the current study, we evaluated the contributors in 4 groups as follows: Group 1: women with non-progressive labor; group 2: women candidate for elective cesarean; group 3: non-pregnant nulliparous women; and group 4: the primiparous women with a minimum duration of 6 weeks interval following vaginal delivery.

**Figure 1:**
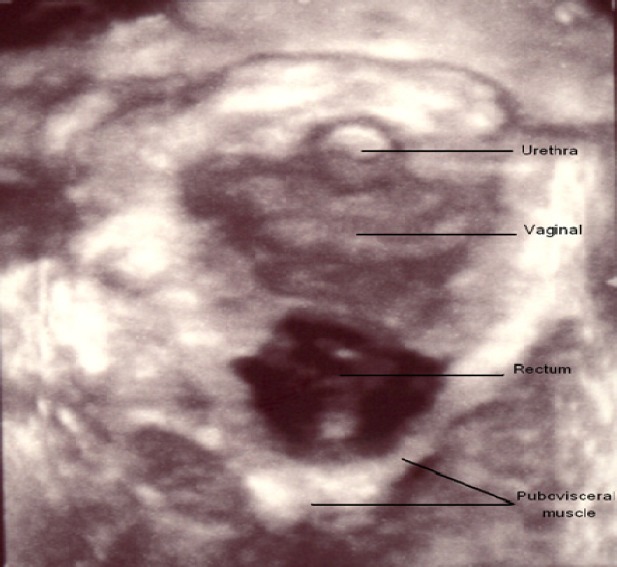
Demonstrates the appearance of the levator hiatus in the axial plane on contraction

This study was approved by the Ethics Committee of Qazvin University of Medical Sciences, Qazvin, Iran. (Ethic No: 28/20/6948).

Continuous variables were checked for normality using graphical methods and are expressed as mean ± SD. Distribution of the diameter and area of pelvic floor were analyzed by ANOVA after testing the normality of measures, and categorical variables were compared using the χ^2^-test and are reported as percentages.

Pearson – ρ was considered for assessing linear correlation.

In some studies, it may be advisable to set a threshold criterion to identify cases to be considered “high” or “low”. When the purpose of the analysis is to compare groups, such as diameters of pelvic floor versus kinds of pregnancy and vaginal delivery, a percentile, such as the 80th percentile for the combined population, can be used as the division point for the dichotomization. Therefore, Comparison of frequencies of participants was performed by test following determining the cut-off value for the 80th percentile. Statistical analysis was performed using SPSS for Windows (version 19; SPSS Inc., Chicago, IL, USA). Also, P-value less than 0.05 were considered as significant differences.

## 3. Results

The mean age of mothers, weight, and gender of babies are shown in Table 1. The weight of babies in elective group was 3.44±0.32 kg which was higher than those in other groups and the difference was found to be significant (P = 0.045).

**Table 1:** Comparison of age, neonates’ gender and babies’ weight in four study groups

Variables		Non-progressive labor	Elective	Nulliparous	NVD		*P*
Age	Mean±SD	24.68±4.12	25.24±4.71	27.04±4.79	23.92±5.31	1.953*	0.126
	(Min,Max)	(18,32)	(18,36)	(20,35)	(17,39)		
Neonate gender	Male N (%)	11(44)	11(44)	-	7(28)	1.799**	0.407
	Female N (%)	14(56)	14(56)	-	18(72)		
Baby weight	mean±SD	3.26±0.39	3.44±0.32	-	3.19±0.39	3.248*	0.045
	(Min,Max)	(2.5,3.8)	(2.8,3.8)	-	(2.5,3.8)		

* =F and

** =χ^2^

The mean BMI of patients in non-progressive labor group was 27.33±2.96, in cesarean elective group 27.48±3.16, in nulliparous group 27.70±2.66, and in NVD group 27.59±3.15 (P = 0.967).

There was no significant correlation between age and the pelvic floor diameter on Valsalva maneuver (P = 0.23; r = -0.124). Also, a significant linear correlation was found between the pelvic floor diameter and the area at three positions of Valsalva maneuver, rest, and contraction (r=0.917, P <0.001, 0.982, <0.001, and 0.933, <0.001) respectively. The pelvic floor diameter and area at three positions in all study groups are shown in table 2.

**Table 2:** Pelvic floor diameter (mm) and area at tree positions in four study groups

Variables	Non-progressive labor (Mean±SD)	Elective (Mean±SD)	Nulliparous (Mean±SD)	NVD (Mean±SD)	*P*
Pelvic floor diameter at rest	214.96 ± 36.90	197.32 ± 32.78	194.95 ± 17.70	206.68 ± 30.46	0.109
Pelvic floor area at rest	34.16 ± 12.52	28.73 ± 9.01	26.17 ± 4.91	30.84 ± 9.55	0.025
Pelvic floor diameter during Valsalva	233.97 ± 47.64	216.36 ± 41.33	206.89 ± 19.63	224.01 ± 36.19	0.116
Pelvic floor area during Valsalva	42.56 ± 21.12	34.04 ± 11.87	29.58 ± 5.67	35.96 ± 11.80	0.012
Pelvic floor diameter during contraction	206.65 ± 35.89	192.25 ± 35.54	180.47 ± 27.58	201.08 ± 32.45	0.057
Pelvic floor area during contraction	31.10 ± 10.81	26.74 ± 9.77	23.06 ± 3.59	29.38 ± 10.32	0.014

The pelvic area during contraction was 31.1±10.81 for non-progressive labor group which was higher than those found for other groups and the difference was significant (P = 0.014), however, the values found for diameter at rest, on valsalva maneuver, and during contraction failed to demonstrate a significant difference. As shown in table 3, based on the 80th percentile for the area and diameter in non progressive labor group at rest, 11 patients had pelvic floor area higher than 36.91. In addition, 8 patients in the same group had a pelvic floor area of higher than 35.28 during contraction and the differences, compared to other groups, were significant (P = 0.004, 0.023, respectively).

**Table 3:** Comparison with clinical percentage of pelvic floor diameter and area at tree positions in four study groups

		Group N (%)				χ^2^	*P*

		Non-progressive labor	Elective	Nulliparous	NVD		
Pelvic floor diameter at rest	<Percentile 80 (226.64)*	16(64)	21(84)	18(92)	21(84)	5.750	0.124
	> Percentile 80 (226.64)	9(36)	4(16)	2(10)	4(16)		
Pelvic floor area at rest	<Percentile 80 (36.91)	14(56)	21(84)	24(96)	21(84)	13.500	0.004
	> Percentile 80(36.91)	11(44)	4(16)	1(4)	4(16)		
Pelvic floor diameter during Valsalva	<Percentile 80 (251.92)	19(76)	19(76)	19(95)	19(76)	3.563	0.313
	> Percentile 80(251.92)	6(24)	6(24)	1(5)	6(24)		
Pelvic floor area during Valsalva	<Percentile 80 (44.23)	19(76)	17(68)	24(96)	21(84)	6.953	0.073
	> Percentile 80(44.23)	6(24)	8(32)	1(4)	4(16)		
Pelvic floor diameter during contraction	<Percentile 80 (222.04)	17(68)	19(76)	20(100)	20(80)	7.500	0.058
	> Percentile 80(222.04)	8(32)	6(24)	0(0)	5(20)		
Pelvic floor area during contraction	<Percentile 80 (35.28)	17(68)	18(72)	25(100)	20(80)	9.500	0.023
	> Percentile 80 (35.28)	8(32)	7(28)	0(0)	5(20)		

* units= millimeter (mm).

Comparing the puborectalis avulsion in non-progressive labor and NVD groups, it was found that 8 and 13 mothers were with avulsion, respectively, nevertheless the difference between the two groups was shown to be insignificant (Table 4).

**Table 4:** Comparison of percentage obtained for puborectalis rupture in four study groups

Variable		Group N (%)		Chi-square	*P*

		Non-progressive labor	NVD		
Puborectalis rupture	no	17 (68)	12 (48)	2.053	0.252
	yes	8 (32)	13 (52)		

Furthermore, we were interested in assessing the association between the puborectalis avulsion and pelvic floor diameter at three positions. In this regard, out of 19 patients with diameters higher than cut-offs at rest and during Valsalva maneuver, 8 patients had puborectalis avulsion (P = 0.025).

## 4. Discussion

The main cause of pelvic floor disorders is not well known but it is considered to be multifactorial. Previous epidemiological studies have suggested different contributing factors including NVD, application of a number of instruments like vacuum and forceps, episiotomy, epidural anesthesia, high maternal age, and other predisposing factors such as previous hysterectomy, aging, menopause, and obesity (Gürel & Gürel, 1999; Carley, Turner, Scott, & Alexander, 1999).

In a study conducted in the United States in 2002, it was shown that increasing parity and obesity may act as the risk factors of pelvic floor disorder (Hendrix et al., 2002).

Muscle and nerve damage of levator ani during vaginal delivery is rumored to be the reason for stretching and wasting of the ligaments in the future. The degree of damage to the connective tissues around the uterus and vagina occurs during labor, but it is usually the vaginal dilation during labor that is considered to be the cause of prolapse (Karasick & Spettell, 1997).

The health stability of pelvic floor is in connection with the healthiness of its structure, function, and interaction of passive and active components (Patel, 2006; Bump & Norton, 1998).

Pelvic floor disorders occur due to the loosening of the support of pelvic organs which is achieved by endopubic fascia and pelvic floor muscles especially the levator ani. Most researchers have described that the NVD can cause direct injury to the supporting infrastructures and indirect injuries to pelvic floor nerves (Richardson, Lyon, & Williams, 1976; Handa, Harris, & Ostergard, 1996).

As a result of nerve injury, the muscle tone decreases and the pressure on uterosacral ligament, parametrium, and endopelvic fascia increases, causing a secondary damage (DeLancey, 1993; Weidner, Barber, Visco, Bump, & Sanders, 2000). During normal pregnancy, the passage of fetal head through the pelvic floor causes significant deformity in muscles and other tissues (Dietz, Wilson, 2005; Parente, Jorge, Mascarenhas, Fernandes, & Martins, 2008). Decrease in the muscle activity after delivery could be detected by common techniques such as digital examination, weight assessment, and perineometry using translabial sonography (Baessler & Schuessler, 2003; Dietz & Shek, 2008).

Nielson et al reported that 34% of women were not able to voluntarily contract their pelvic floor muscle up to 6 weeks after delivery. Imaging studies have shown that up to 20% of women with natural delivery have defects mostly in the levator ani in pubovisceral parts in the majority of cases (DeLancey, Kearney, Chou, Speights, & Binno, 2003; Dietz & Lanzarone, 2005).

The increase in genital hiatus occurring following natural delivery is related to the prolapse of pelvic organs (Delancey & Hurd, 1998; Panayi & Khullar, 2009).

In the present study, the pelvic floor area in women with non-progressive labor was 34.16±12.52 at rest, 42.56±21.12 on Valsalva maneuver, and 31.10±10.81 during contraction. Also, the pelvic floor area was significantly higher in women with non-progressive labor at rest (P = 0.025), on Valsalva maneuver (P = 0.012), and during contraction (P = 0.014) than in nulliparous women.

## 5. Conclusion

Previous studies provide data on assessing all sonographic parameters by 3–4D introital ultrasonography which prompted us to objectify the anatomical changes in pelvic floor constructions due to pregnancy and delivery. These have showed to be significantly different in comparison to nulliparous women. Irreversible traumatic overdistension of the levator hiatus or ‘microtrauma’ may be an additional form of pelvic floor injury related to childbirth. During vaginal delivery, the pelvic floor is distinctly distended by the fetal head, which may possibly lead to vascular, neuromuscular, and connective tissue changes. Our findings indicate that the unilateral avulsion of the puborectalis muscle results in the asymmetry of levator hiatus and hiatal diameter.

There were 21 (42%) cases of puborectalis avulsion observed in the non-progressive labor group (8) and NVD group (13). The results showed that non-progressive labor is one of the important risk factors for the occurrence of pathological changes in women’s pelvic muscles and this highlights the necessity for a better management and application of a timely cesarean in women susceptible or at risk of non-progressive labor.
